# Elements of Modern Chemistry

**Published:** 1879-07

**Authors:** 


### BIBLIOGRAPHICAL.

Elements of Modern Chemistry.—By Adolph Wurtz, member of the Institute, Honorary Dean and Professor of Chemistry of the Faculty of Paris, etc. Translated and edited, with approbation of the author, from the Fourth French edition. By Wm. H. Green, M.D., formerly Demonstrator of Chemistry in Jefferson Medical College, etc; with one hundred and thirty-two illustrations. J. B. Lippincott & Co. Philadelphia–1879.

The great difficulty experienced with most persons in the study of chemistry is the complexity of its principles and the multiplicity of its details. Whole chapters might be studied on the reactions of chemicals and the resulting compounds and the mind retain but a confused idea of the *modus operandi* of the processes, or the physical and chemical properties of the substances. When, however, the laws of definite proportions or equivalents of atomic composition; of isomorphism of isomerism and of chemical momenclature and notation are thoroughly understood the difficulties of the study are to a considerable extent, dissipated. These fundamental principles are expounded and expatiated upon in the introductory part of the work before us, and we think made as clear as it is possible to make them in writing.

The usual arrangement as to the composition and general properties of metalic compounds is next presented, and very little difference is noticed in this department to what we find in other books on chemistry. The classification of the metals as to their varied affinities and capacities of combination is full and comprehensive, and adds much to the value of the book. The space devoted to organic chemistry is sufficient to permit a comprehensive presentation of this part of the study, and will recommend it to students. The most serious objection that we have met with in perusing the work is the absence of a table of contents. Those to whom the book is particularly suitable otherwise, will find this defect almost irreparable.

